# Weakly activated core neuroinflammation pathways were identified as a central signaling mechanism contributing to the chronic neurodegeneration in Alzheimer’s disease

**DOI:** 10.3389/fnagi.2022.935279

**Published:** 2022-09-27

**Authors:** Fuhai Li, Abdallah M. Eteleeb, William Buchser, Christopher Sohn, Guoqiao Wang, Chengjie Xiong, Philip R. Payne, Eric McDade, Celeste M. Karch, Oscar Harari, Carlos Cruchaga

**Affiliations:** ^1^Institute for Informatics (I2), Washington University in St. Louis School of Medicine, St. Louis, MO, United States; ^2^Department of Pediatrics, Washington University in St. Louis School of Medicine, St. Louis, MO, United States; ^3^NeuroGenomics and Informatics, Washington University in St. Louis School of Medicine, St. Louis, MO, United States; ^4^Department of Psychiatry, Washington University in St. Louis School of Medicine, St. Louis, MO, United States; ^5^Hope Center for Neurological Disorders, Washington University in St. Louis School of Medicine, St. Louis, MO, United States; ^6^Department of Neuroscience, Washington University in St. Louis School of Medicine, St. Louis, MO, United States; ^7^Division of Biostatistics, Washington University in St. Louis School of Medicine, St. Louis, MO, United States; ^8^Department of Neurology, Washington University in St. Louis School of Medicine, St. Louis, MO, United States

**Keywords:** neuroinflammation, signaling network, Alzheimer’s disease, molecular mechanism, signaling targets

## Abstract

**Objectives:**

Neuroinflammation signaling has been identified as an important hallmark of Alzheimer’s disease (AD) in addition to amyloid β plaques (Aβ) and neurofibrillary tangles (NFTs). However, the molecular mechanisms and biological processes of neuroinflammation remain unclear and have not well delineated using transcriptomics data available. Our objectives are to uncover the core neuroinflammation signaling pathways in AD using integrative network analysis on the transcriptomics data.

**Materials and methods:**

From a novel perspective, i.e., investigating weakly activated molecular signals (rather than the strongly activated molecular signals), we developed integrative and systems biology network analysis to uncover potential core neuroinflammation signaling targets and pathways in AD using the two large-scale transcriptomics datasets, i.e., Mayo Clinic (77 controls and 81 AD samples) and ROSMAP (97 controls and 260 AD samples).

**Results:**

Our analysis identified interesting core neuroinflammation signaling pathways, which are not systematically reported in the previous studies of AD. Specifically, we identified 7 categories of signaling pathways implicated on AD and related to virus infection: immune response, x-core signaling, apoptosis, lipid dysfunctional, biosynthesis and metabolism, and mineral absorption signaling pathways. More interestingly, most of the genes in the virus infection, immune response, and x-core signaling pathways are associated with inflammation molecular functions. The x-core signaling pathways were defined as a group of 9 signaling proteins: MAPK, Rap1, NF-kappa B, HIF-1, PI3K-Akt, Wnt, TGF-beta, Hippo, and TNF, which indicated the core neuroinflammation signaling pathways responding to the low-level and weakly activated inflammation and hypoxia and leading to the chronic neurodegeneration. It is interesting to investigate the detailed signaling cascades of these weakly activated neuroinflammation signaling pathways causing neurodegeneration in a chronic process, and consequently uncover novel therapeutic targets for effective AD treatment and prevention.

**Conclusions:**

The potential core neuroinflammation and associated signaling targets and pathways were identified using integrative network analysis on two large-scale transcriptomics datasets of AD.

## Introduction

A major challenge limiting effective treatments for Alzheimer’s disease (AD) is the complexity of AD. More than 42 genes/loci have been associated with AD (Sims et al., 2017; Verheijen and Sleegers, 2018). Unfortunately, only few of these genes, such as *CD33* (Zhao, 2019), *TREM2* (Gratuze et al., 2018), and *MS4A* (Deming et al., 2019), are being evaluated as therapeutic targets for AD management (Verheijen and Sleegers, 2018). Over 240 drugs have been tested in AD clinical trials, but only one drug, i.e., Aduhelm (aducanumab) was recently approved for the treatment of AD since 2003 (Alzheimer’s Association, 2018; Cummings et al., 2018; Dunn et al., 2021). One major challenge is that the complicated pathogenesis and core signaling pathways of AD remain unclear. Therefore, it is significant to uncover the core signaling pathways implicated on AD pathogenesis and novel therapeutic targets of AD for identifying effective drugs and synergistic drug combinations (targeting multiple essential targets on the cores signaling network) for AD prevention or treatment. Our knowledge of the molecular mechanisms and signaling pathways that ultimately lead to the chronic neurodegeneration in AD is limited. For example, there are only a few strong genetic biomarkers for AD that have been identified, including the *APOE*, *APP*, and *PSEN1/2* genes. However, the signaling consequence of these biomarkers, as they relate to the accumulation of dysfunctional A-beta and p-Tau proteins, as well as neuron degeneration and immune response, remains unclear.

Over the last 10 years, neuroinflammation signaling has been identified as the third core feature or a central pathogenesis mechanism of AD (Akiyama et al., 2000; Combs et al., 2000; Ekdahl et al., 2003; Kinney et al., 2018; Knezevic and Mizrahi, 2018; Newcombe et al., 2018), in addition to amyloid β plaques (Aβ) and neurofibrillary tangles (NFTs) pathologies. The detailed occurrence and roles of neuroinflammation are complex and remain unclear, though set of inflammation and immune genes, such as TNF (Decourt et al., 2017), IL-1beta (Shaftel et al., 2008; Ng et al., 2018), IL-6 (Ng et al., 2018), and NFkB (Chen et al., 2012), have been reported (Guzman-Martinez et al., 2019). For example, the accumulation of altered proteins, such as the Aβ and tau protein, are toxic to neuron cells and are believed to trigger the neurodegeneration diseases (Guzman-Martinez et al., 2019). Not restricted to the neurons, the neuroinflammation and immune response are reported to be regulated and affected by the neuron-immune cell interactions (Heneka et al., 2015; Leng and Edison, 2021), such as macroglia and astrocytes(Gratuze et al., 2018; Shi et al., 2019; Mahan et al., 2022). Moreover, peripheral infections are also identified to be associated with degeneration diseases (McManus and Heneka, 2017).

On the other hand, large-scale transcriptomics datasets of AD and control samples have been generated, such as ROSMAP (Bennett et al., 2018; de Jager et al., 2018) and Mayo Clinic (Allen et al., 2016), to investigate the essential targets and signaling pathways in AD. Network analysis models were proposed to identify the potential dysfunctional signaling pathways and biomarkers using these related RNA-seq datasets. For example, the molecular signatures and networks under different brain regions were reported using integrative co-expression network analysis, and the myelin signaling dysregulation was identified in AD (Wang et al., 2016; Wan et al., 2020). In addition, the co-splicing network using the WGCNA (co-expression network analysis model) was conducted to identify the altered splicing in AD, which indicated that the altered splicing is the mechanism for the effects of the AD-related CLU, PTK2b, and PICALM alleles (Raj et al., 2018). Moreover, the molecular subtypes and potential driver genes, such as CABRB2, LRP10, and ATP6V1A of AD, were identified by combing key driver analysis (KDA) and multiscale embedded gene expression network analysis (MEGENA) (McKenzie et al., 2017; Neff and Vatansever, 2020; Neff et al., 2021). Moreover, the meta-analysis of multi-datasets of human and mouse AD samples has been studied to understand the essential biomarkers and signaling modules of AD (Wan et al., 2020).

However, the systematic delineation of core neuroinflammation signaling pathways has not been reported yet in these previous computational data analysis models using the transcriptomics data (Guzman-Martinez et al., 2019). Therefore, it is important to continue to pursue systematic investigations, including the use of network analysis techniques, in order to understand the details of core neuroinflammation and immune signaling pathways that are associated with the neurodegeneration of AD. In response to the preceding gap in knowledge, herein, we systematically sought to identify the potential core neuroinflammation and related signaling pathways that potentially cause neuron degeneration in AD by analyzing the large-scale transcriptomics, i.e., RNA-seq data of human AD samples (Bennett et al., 2018; de Jager et al., 2018). Inspired by the observation of that strongly activated (hyper-) inflammation and immune (Catanzaro et al., 2020; Patel et al., 2021) signaling pathways are often associated with relatively faster/acute disease progression, such as what is observed in COVID-19 (Gordon et al., 2020; Li et al., 2020) here we hypothesize that the neuroinflammation in AD (a chronic disease) is weakly activated (i.e., with small fold change values compared with control samples). In another word, the transcriptomics changes in neuroinflammation are at a low level, which might be missed by only detecting the strongly activated signaling targets and pathways. This hypothesis was also supported by the that there are only a few strongly activated signals in AD samples vs. control, which is defined as large gene expression fold-change, e.g., fold-change ≥2.0 (see the results) (Andrews, 2010; Dobin et al., 2013; Patro et al., 2017; Gordon et al., 2020; Li et al., 2020). Specifically, we employed the RNA-seq data of neuropathology-free controls and AD samples from two datasets: ROSMAP (Bennett et al., 2018; de Jager et al., 2018) and Mayo Clinic (Allen et al., 2016). Leveraging these data, we then identified all of the weakly activated and inhibited genes with very low fold-change thresholds. Subsequently, we conducted network analyses to identify relevant core neuroinflammation signaling pathways. More importantly, the potential core neuroinflammation and associated signaling pathways were identified using large-scale transcriptomics datasets.

## Materials and methods

### Gene expression data and analysis of Alzheimer’s disease samples

In this study, 77 control tissue samples and 81 AD pathological aging samples in Mayo dataset^1^; and 97 control samples and 260 AD dorsolateral prefrontal cortex samples in ROSMAP dataset^2^ that passed the quality control using FastQC (Andrews, 2010) were used. The AD and control samples in Mayo dataset were identified using the diagnosis result in the clinical data. The definition of AD and control cases was provided in the study description, which is available at.^3^ As introduced, two experts, i.e., Dr. Dennis Dickson or Dr. Thomas Beach, conducted the neuropathologic evaluation of Mayo samples, respectively. The AD cases were diagnosed according to the NINCDS–ADRDA criteria and with Braak NFT stage of IV or greater. The control cases had Braak NFT stage of III or less; and the CERAD neuritic and cortical plaque densities are less than 1 (1 represents “sparse” and 0 means “none”) and had no any of the dementia-related diseases. The clinical and neuropathological data available from the ROSMAP cohort were used to classify controls and AD cases in our analyses. Specifically, subjects that exhibit no decline in cognition and scored Low or No AD on the Reagan scale, and with a BraakTau score of ≤3, were identified as controls. The AD cases were identified as patients who exhibit clear cognition decline as well as scored high or intermediate on the Reagan scale (Bennett et al., 2018). For RNA-seq data analysis, these RNA-seq data were first aligned to reference genome GRCh38 using STAR (v.2.7.1a) (Dobin et al., 2013). We excluded ALT, HLA, and Decoy contigs from the reference genome due to the lack of RNA-Seq tools that allow to handle these regions properly. Then, reads alignment was further evaluated by applying Picard CollectRnaSeqMetrics^4^ based on the GENCODE^5^ annotations to examine reads distribution on the genome. Samples that showed a Percent Duplication of >50% were flagged for removal. Then, the transcripts per million (TPM) values of 16,132 common protein-coding genes were then obtained in the two datasets by applying the Salmon quantification tool (Patro et al., 2017) in alignment-based mode using the aligned RNA-seq data using the coding transcripts of the reference genome.

### Differentially expressed genes

To identify the upregulated and downregulated genes in AD samples vs. control samples, the limma-voom (Law et al., 2014; Ritchie et al., 2015) approach, taking the normalized [using trimmed mean of M values (TMM) (Robinson and Oshlack, 2010)] log-transformed TPM values as the input, in edgeR (Robinson et al., 2010) R package, was employed.

### Inflammation genes

A set of 1,043 inflammation genes were obtained by extracting genes from the inflammatory response category as defined in the Gene Ontology (GO:0006954) (Gene Ontology Consortium et al., 2000). Subsequently, 485 inflammation genes were obtained from the 5,191 kyoto encyclopedia of genes and genomes (KEGG) signaling genes, i.e., the interaction between the KEGG signaling pathways and GO inflammation genes.

### Alzheimer’s disease GWAS data

The GWAS data of AD was obtained from NIAGADS database (Kunkle et al., 2019).^6^ The Stage 1 *p*-Value Data (updated by 26 February 2019) and Stage 2 *p*-Value Data (updated by 27 February 2019) were downloaded. The 553 candidate GWAS genes and also available in the KEGG signaling pathways were obtained by a filter with *p*-value ≤ 1.0 × 10^–5^.

### Kyoto encyclopedia of genes and genomes signaling pathway enrichment analysis

The KEGG signaling pathways were extracted using the graphite R package (Sales et al., 2012), which consists of 311 signaling pathways (Ogata et al., 1999; Kanehisa and Goto, 2000). There are 59,242 signaling interactions among 5,191 genes in these pathways, which were used for network enrichment analysis and network inference analysis in this study. For the network enrichment analysis, a Fisher’s exact test (Fisher, 1932; Kim, 2017) was used based upon the upregulated genes.

### KEGG signaling network inference analysis

To infer the signaling cascades among a set of genes of interest, we developed a network inference approach. First, we divided the genes into two groups: signaling sources (like the inflammation signaling genes) and signaling targets (like the apoptosis signaling genes). Second, a signaling network was constructed by linking the signaling source genes to the signaling target genes iteratively. Specifically, the signaling source genes were used as the initial signaling source nodes set: V0. The signaling target genes were used as the target nodes set: V1. In the iterative process, the shortest signaling cascades/paths between the nodes in V0 and V1 were calculated and identified: *P_*ij*_* ≤ *g_*i*_, g_*k*1_, g_*k*2_*,…, *g_*j*_* >, *where g_*i*_ belongs to V0*, and *g_*j*_ belongs to V1*. Third, all of the genes on the signaling path *P*_*ij*_ and belong to V1 were selected and added to V0, and removed from V1. This process was repeated until all the genes were added to V0. The data analysis codes are available upon request.

## Results

### Normal and Alzheimer’s disease tissue samples are barely separable in the gene expression data space

**FIGURE 1:**
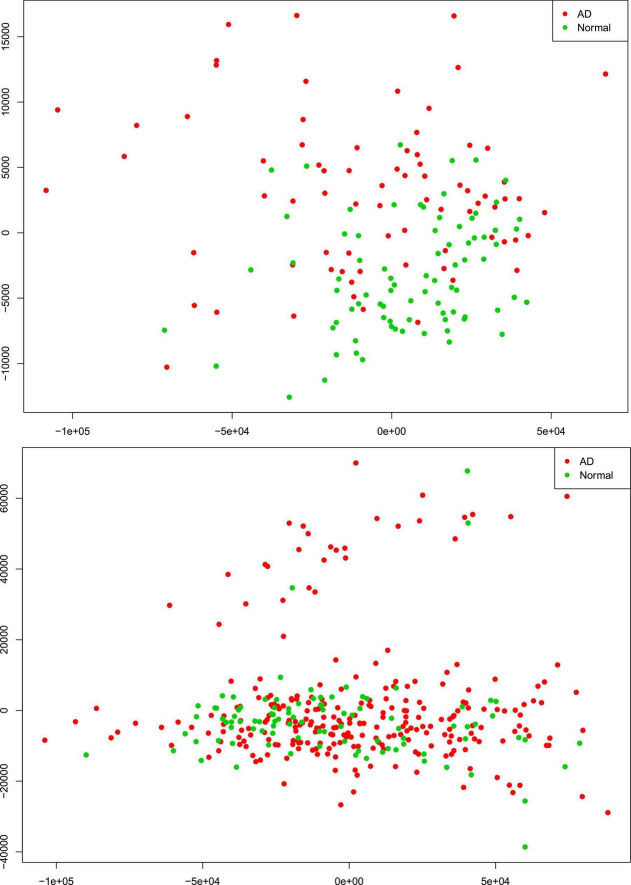
Alzheimer’s disease (AD) and control tissue samples are not well separable in the first (*x*-axis) and second (*y*-axis) principal components of MDS analysis on the RNA-seq protein-coding genes in Mayo (top-panel) and ROSMAP (bottom-panel) datasets.

There were 77 control subjects and 81 AD cases in Mayo dataset; and 97 control samples and 260 AD cases in ROSMAP dataset. Table 1 shows the epidemiology information of Mayo and ROSMAP datasets. The transcripts per million (TPM) values of 16,132 protein-coding genes were obtained by applying the Salmon quantification tool (Patro et al., 2017) in alignment-based mode using the STAR-aligned RNA-seq data. A multidimensional scaling (MDS) model was used to generate the 2D clustering plots of control and AD samples in the Mayo and ROSMAP datasets, respectively (see Figure 1). As seen in these visualizations, the control and AD samples are barely separable, especially in the ROSMAP dataset, which of note, has more control samples than Mayo dataset.

**TABLE 1:** Epidemiology information of Mayo and ROSMAP datasets.

Mayo	Control	AD	ROSMAP	Control	AD
Total	77	81	Total	97	260
Male	40	33	Male	44	82
Female	37	48	Female	53	178
Age, mean (SD)	82.65 (8.70)	82.57 (7.62)	Age, mean (SD)	84.24 (6.82)	90.34 (5.75)
Braak, mean (SD)	2.18 (0.86)	5.53 (0.52)	Braak, mean (SD)	1.88 (0.98)	4.25 (0.85)
APOE_22	0	0	APOE_22	2	0
APOE_23	12	4	APOE_23	13	22
APOE_33	56	34	APOE_33	72	141
APOE_24	1	0	APOE_24	1	10
APOE_34	8	36	APOE_34	8	83
APOE_44	0	7	APOE_44	1	3

We further conducted a widely used differential expression analysis method to identify differentially expressed genes (DEGs) between the AD and control samples. To identify the common set of DEGs between the two datasets, we applied a number of fold-change and *p*-value thresholds. As seen in Supplementary Table 1 and expected from Figure 1, only a few common upregulated and downregulated DEGs were identified with fold change thresholds ≥1.5 and *p*-value ≤ 0.05. With the fold-change threshold ≥1.25, only about 230 upregulated and about 60 downregulated genes were identified (out of the 16,132 protein-coding genes, ∼1.85%), in both studies. When relaxing both thresholds to fold change ≥1.1 and *p*-value ≤ 0.1, 1,120 upregulated genes and 689 downregulated genes were identified (∼11.2% of the 161,32 protein-coding genes). Based on these observations, we hypothesized that the AD-associated signaling pathways are weakly activated or inhibited. The “weakly” term is defined as that the differentially expressed genes have small fold-change values.

### Weak inflammation and hypoxia are the potential major factors in the Alzheimer’s disease brain microenvironment causing neuron cell death

**FIGURE 2:**
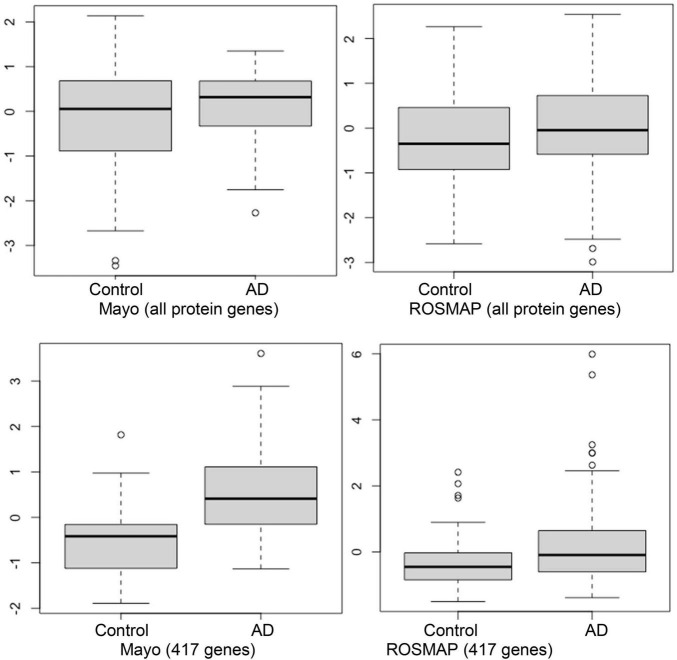
Box plots of the first principal component of MDS analysis in control and AD cases. **Left and right columns** are Mayo and ROSMAP samples, respectively. **Upper and lower panels** represent the MDS analysis using all genes and 417 upregulated genes, respectively. *Y*-axis is the first principal component of MDS analysis.

As was noted previously, we believe it is important to identify AD-associated weakly activated signaling pathways and understand their roles in AD disease progression, as well as their potential roles as targets for AD therapeutics. Among the 1,120 common upregulated genes (identified from Mayo and ROSMAP datasets), 417 genes were included in the 311 KEGG signaling pathways, and the rest genes were not available in the KEGG signaling pathways. To this end, we first conducted an enrichment analysis of KEGG signaling pathways using Fisher’s exact test applied to the 417 upregulated genes. Table 2 showed the enriched signaling pathways with *p*-value ≤ 0.15. We then clustered these activated signaling pathways empirically into 7 categories. Using these 417 upregulated genes, the first principal component values in the MDS analysis of the AD and control samples were used to compare the difference between AD and control samples. The OR, absolute beta values, and *p*-values of logistic regression analysis (see Table 3) indicated that these selected genes [*p*-value = 1.22 × 10^–13^ (Mayo) and *p*-value = 4.2 × 10^–6^ (ROSMAP)] can separate the AD and control samples much better than using all protein genes [*p*-value = 0.036 (Mayo) and *p*-value = 0.027 (ROSMAP)] in the two datasets, respectively. The box plots are also provided in Figure 2, which indicated that the control and AD samples are more separable using the selected genes.

**TABLE 2:** Upregulated genes in TNF and apoptosis signaling pathways.

Apoptosis	BCL2, RELA, BIRC3, FADD, GADD45G, TNFRSF1A, NFKBIA, TNFRSF10B, CAPN2, TUBA1C, IL3RA, CTSH, FOS, CASP6, CASP7, TNFRSF10A, PARP4
TNF signaling pathway	RELA, BIRC3, FADD, MAP2K3, TNFRSF1A, NFKBIA, CREB3L2, FOS, CASP7, MLKL, IRF1, CEBPB

**TABLE 3:** Odds ratio (OR), beta, and *p*-values of logistic regression using all genes and 417 upregulated genes.

	All genes			417 genes		
		
	OR	abs(beta)	*P*-value	OR	abs(beta)	*P*-value
Mayo	1.42	0.35	0.037	5.9	1.78	9.5 × 10^–9^
ROSMAP	1.31	0.27	0.027	1.9	0.63	9.7 × 10^–5^

**FIGURE 3:**
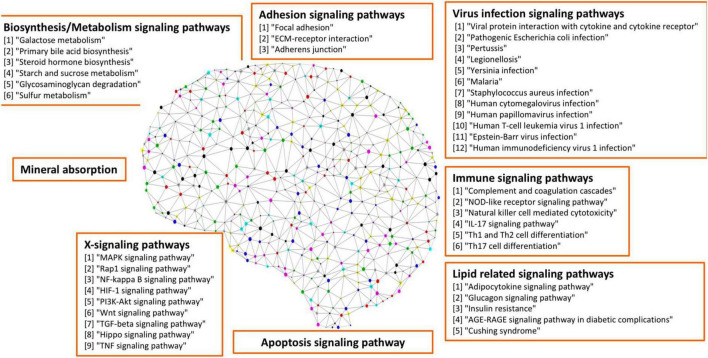
Seven categories of weakly activated signaling pathways in AD.

As seen in our results (see Figure 3 and Table 4), a set of signaling pathways were activated, such as those involved in virus infection signaling [including Epstein-Barr virus, Human T-cell leukemia virus 1 infection, Legionellosis, Pathogenic *Escherichia coli* infection, *Staphylococcus aureus* infection, Yersinia infection, Human cytomegalovirus infection, Human papillomavirus infection, Malaria, Human immunodeficiency virus 1 infection, Rheumatoid arthritis, and Inflammatory bowel disease (IBD)]. Out of the 417 upregulated genes, 111 genes were in common across these pathways highlighting a set of core genes implicated on these processes. These results indicated that weakly activated inflammation-related signaling pathways, such as inflammation, cytokine, and immune response, may be represent activated signaling pathways in the AD brain microenvironment.

**TABLE 4:** The seven categories of enriched kyoto encyclopedia of genes and genomes (KEGG) signaling pathways.

Name	*P*-value	Name	*P*-value
**Virus-related signaling pathways**		**x-core signaling pathways**	
Viral protein interaction with cytokine and cytokine receptor	0.0019	PI3K-Akt signaling pathway	0.0011
Epstein-Barr virus infection	0.0056	MAPK signaling pathway	0.0059
Human T-cell leukemia virus 1 infection	0.0188	NF-kappa B signaling pathway	0.0085
*Staphylococcus aureus* infection	0.0249	Hippo signaling pathway	0.0132
Human papillomavirus infection	0.0299	TGF-beta signaling pathway	0.0137
Pertussis	0.0375	TNF signaling pathway	0.0434
Yersinia infection	0.0397	Rap1 signaling pathway	0.0571
Pathogenic *Escherichia coli* infection	0.0430	HIF-1 signaling pathway	0.1009
Human cytomegalovirus infection	0.0603	Wnt signaling pathway	0.1043
Malaria	0.0758	Apoptosis	0.0658
Legionellosis	0.0906		
Human immunodeficiency virus 1 infection	0.1075		
Rheumatoid arthritis	0.1192	**Mineral absorption**	2.56E-05
Inflammatory bowel disease (IBD)	0.1321		
**Immune signaling pathways**		**Diabetic/Lipid signaling pathways**	
IL-17 signaling pathway	0.0104	AGE-RAGE signaling pathway in diabetic complications	0.0021
Complement and coagulation cascades	0.0214	Adipocytokine signaling pathway	0.0060
NOD-like receptor signaling pathway	0.0401	Insulin resistance	0.0283
Th17 cell differentiation	0.1275	Glucagon signaling pathway	0.1179
Th1 and Th2 cell differentiation	0.1368	Cushing syndrome	0.1356
Natural killer cell-mediated cytotoxicity	0.1410		
**Biosynthesis/Metabolism signaling pathways**		**Adhesion signaling pathways**	
Sulfur metabolism	0.0758	Focal adhesion	1.39E-05
Galactose metabolism	0.0812	ECM-receptor interaction	0.0002
Glycosaminoglycan degradation	0.0905	Adherens junction	0.0669
Steroid hormone biosynthesis	0.1084		
Starch and sucrose metabolism	0.1084		
Primary bile acid biosynthesis	0.1437		

**FIGURE 4:**
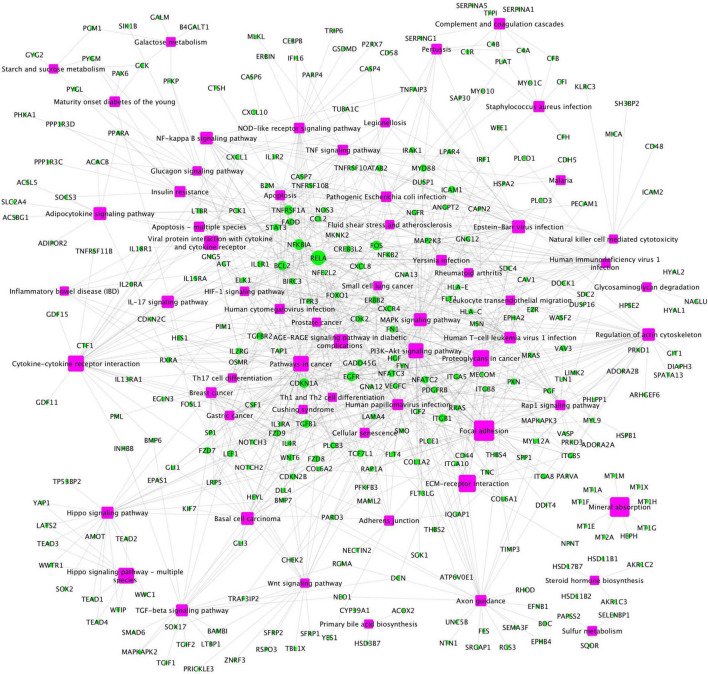
The upregulated gene (green circles)-pathway (purpose squares) interaction network, including 1,021 interactions between 291 upregulated genes and 61 enriched pathways.

In addition, a group of activated signaling pathways or factors that are not clustering to a specific biological function or disease (referred to as the x-signaling pathway: the Hippo, PI3K-Akt, AGE-RAGE, MAPK, Adipocytokine, NF-kappa B, IL-17, TGF-beta, NOD-like receptor, TNF, Apoptosis, HIF-1, and Wnt signaling pathways, as well as apoptosis signaling) were identified. Figure 4 shows the associations between these upregulated genes and activated signaling pathways. As seen in Figure 4, a set of genes in the center areas of the network are associated with a set of signaling pathways, which could represent therapeutic signaling targets that could be used to inhibit or otherwise perturb these activated signaling pathways. In addition, there are a number of metabolisms signaling pathways, such as sulfur metabolism, galactose metabolism, starch and sucrose metabolism, steroid hormone biosynthesis, and glycosaminoglycan degradation, implicated in this model. Moreover, Th1/2/17 (T helper, CD4 + cells) cell differentiation and natural killer cell-mediated cytotoxicity signaling pathways were activated. Supplementary Table 2 lists these associated upregulated genes and the involved signaling pathways. All the observations suggest a potential novel hypothesis that the inflammation, immune signaling, and hypoxia signaling in AD microenvironment activated the *MAPK*, *PI3K-Akt*, and *mTOR* signaling pathways, and then activated the *HIF-1* signaling pathway. However, the activation of *HIF-1* may fail to bring enough oxygen to protect against hypoxic injury to the involved neurons. The dysfunction of blood vessel functions, leading to hypoxia, might be partially indicated by the recent study showing that blood and cerebrospinal fluid flow cleaning the brain during sleeping (Fultz et al., 2019).

### Weak inflammation and hypoxia are the major factors in the Alzheimer’s disease brain microenvironment causing neuron cell death

As was introduced above, many genes that are activated as a function of virus infection, immune response, and the x-core signaling pathways are inflammation-related genes. It is well known that virus infection and immune response signaling pathways respond to inflammation. Our analyses identified 1,043 inflammation response genes in the gene ontology (GO) database (GO:0006954), which includes 492 genes in the KEGG signaling pathways. Interestingly, among the 417 upregulated genes, 66 genes were inflammation related. The *p*-value of observing the 66 upregulated inflammation signaling targets from 417 upregulated genes identified in the AD vs. control samples was 8.34E-05 (calculated using Fishers’ exact test), and the odds ratio (OR) = 1.77, which indicates that the activation of inflammation signaling is concomitant with AD progression. Furthermore, there are 66 overlapping upregulated genes spanning the virus infection (from 111 upregulated genes) and x-core signaling pathways (from 136 upregulated genes), which indicate that the x-core signaling pathways are the likely pathways being activated in response to this inflammation. In addition, the activation of HIF-1 signaling pathway indicates the presence of hypoxia in the AD brain environment.

**FIGURE 5:**
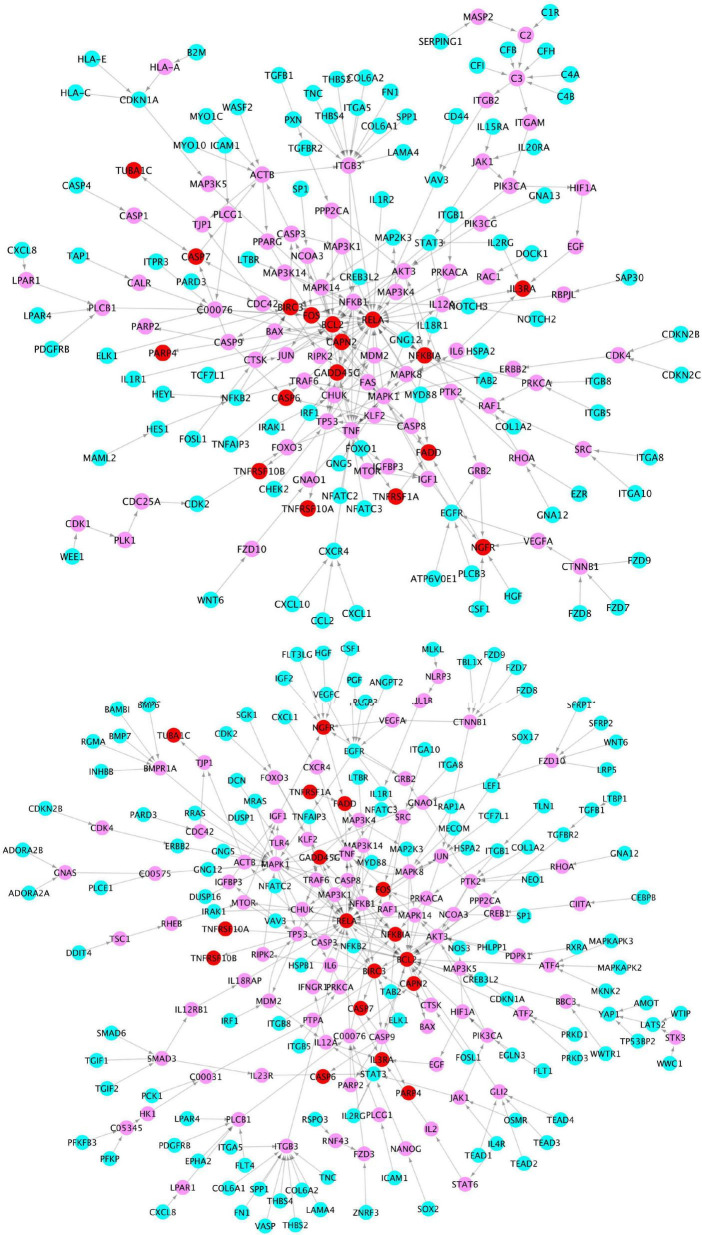
Signaling cascades that links the upregulated genes in the virus infection pathways (cyan) **(top)** and x-core signaling pathways **(bottom)**, respectively, to the upregulated apoptosis signaling genes (red) *via* the linking genes (pink).

To further investigate the network signaling cascades involving inflammation and apoptosis genes, we conducted the network analysis incorporating the activated signaling pathways and apoptosis signaling genes. Figure 5 shows the inferred signaling network that links the upregulated inflammation-related genes (cyan) in virus infection and x-core signaling pathways, respectively, to the apoptosis signaling genes (red). Among the 338 signaling network genes in Figure 5, there were 18 reported GWAS genes (with *p*-value ≤ 1.0 × 10^–5^): *PIK3CB*, *AKT3*, *RAF1*, *MAPK10*, *PPP2R2B*, *ERBB4*, *MECOM*, *IL1R1*, *MYD88*, *CAMK2D*, *GNB4*, *VAV3*, *PRKD3*, *PRKCE*, *THRB*, *FN1*, *LTBP1*, and *WWTR1*, which were reported in the GWAS analysis (Kunkle et al., 2019). The signaling network might indicate the roles of GWAS genes in neuroinflammation and neurodegeneration. For example, the PIK3CB, AKT3, MAPK10, and ERBB4 genes are in the x-core signaling pathways; IL1R1 and MYD88 are cytokine, inflammation, and immune response genes; CAMK2D belongs to serine/threonine protein kinase and Ca2 + protein kinase subfamily. MECOM is involved in apoptosis and is a transcription factor that interacts with SMAD3 and MAPK genes. The GNB4, PRKD3, and PRKCE are related to the cAMP response element-binding protein signaling, which is associated with AD (Saura and Valero, 2011).

**FIGURE 6:**
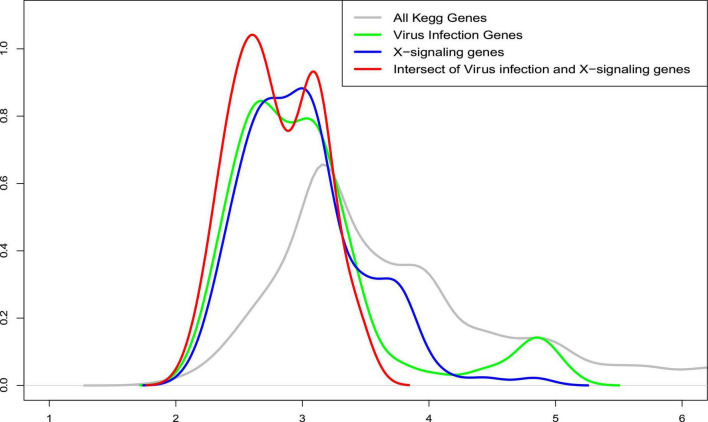
The distribution of average shortest distance on the kyoto encyclopedia of genes and genomes (KEGG) signaling network between the upregulated genes in the inflammation related signaling pathways, including virus infection, immune response, and x-core signaling pathways to the apoptosis signaling genes. As seen, the inflammation signaling genes are much closer (see green, blue, and red) to the apoptosis genes compared with all other signaling genes (see gray lines). *X*-axis represents the shortest path distance; and *y*-axis is the density function.

Further, we also compared the distance distribution among the inflammation-related upregulated genes and apoptosis genes as shown in Figure 6. As can be seen, the inflammation signaling genes are much closer, based on the shortest path metric calculated using Dijkstra’s algorithm, on the signaling network (see green, blue, and red nodes) to the apoptosis genes compared with other signaling genes (see gray lines). These results indicate a potential signaling interaction between the inflammation signaling genes and apoptosis signaling. In other words, the results suggest a potential association that the weak inflammation and hypoxia signaling in the AD brain environment led to chronic neurodegeneration process *via* the activation of the x-core signaling pathways. Therefore, drugs and drug combinations that can perturb the x-core signaling pathways have the potential to be effective for AD prevention and treatment.

### Activated tumor necrosis factor signaling might lead the programmed apoptosis of neurons

**FIGURE 7:**
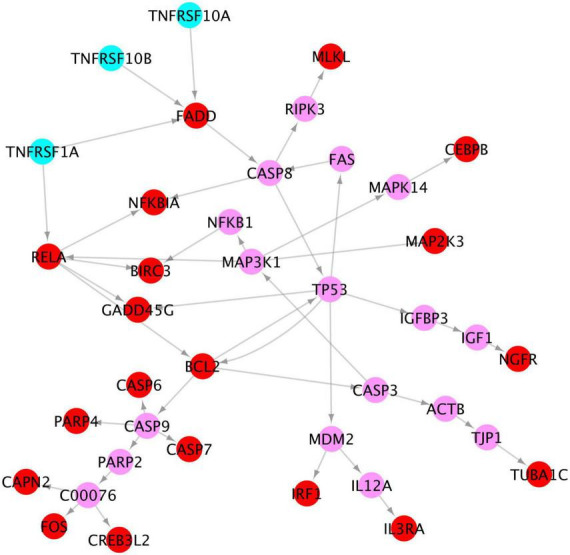
Signaling cascades, causing neuron degeneration, from the 3 TNF receptors (cyan) to the upregulated genes (red) in TNF and apoptosis signaling pathways *via* the linking genes (pink).

Of note, our results show that among the x-core signaling pathways, the TNF signaling pathways are also activated. Particularly, the TNF (Tumor Necrosis Factor) receptors (TNFRSF1A TNFRSF10A, and TNFRSF10B) were upregulated. We reconstructed these signaling pathways linking the TNF receptors to the upregulated genes in TNF and apoptosis signaling pathways (see in Figure 7) using the proposed KEGG network inference model. As seen, the activation of these TNF signaling pathways might be one possible molecular mechanism causing the activation of apoptosis signaling *via* the CASP6 and CASP7 cascades.

## Discussion and conclusion

In addition to Aβ and NFTs, neuroinflammation has been identified as another important AD pathogenesis (Heneka et al., 2015; Shi et al., 2017; Kinney et al., 2018). A set of inflammation and immune genes, such as *TREM2* (Guerreiro et al., 2012; Jonsson et al., 2012), TNF (Decourt et al., 2017), IL-1beta (Shaftel et al., 2008; Ng et al., 2018), IL-6 (Ng et al., 2018), and FkB (Chen et al., 2012) have been reported (Guzman-Martinez et al., 2019). The neural–immune interactions were also reported to exacerbate the AD disease progress (Heneka et al., 2015; Shi et al., 2017; Kinney et al., 2018). Also, the analysis in the human brain endothelial cells and in Alzheimer’s brain, the JNK-AP1 signaling pathway was identified that mediates the activation and expression of inflammatory genes induced by beta-amyloid peptides (Vukic et al., 2009). The network analysis models were proposed to identify the potential dysfunctional signaling pathways and biomarkers using the same RNA-seq datasets (Wang et al., 2016; McKenzie et al., 2017; Raj et al., 2018; Neff and Vatansever, 2020; Wan et al., 2020; Neff et al., 2021). However, neuroinflammation signaling pathways have not been systematically uncovered and analyzed in these reported computational models using the same datasets. Compared with existing studies, the unique contributions are the discovery of systematic core neuroinflammation signaling pathways in AD by investigating the weakly activated molecular signals. We generated supportive evidence of linking these targets with neuroinflammation using enrichment analysis, GO terms, and network analysis systematically. The neuroinflammation signaling pathways, including the virus infection, immune response, x-core signaling pathways, and apoptosis signaling pathways indicated potentially novel targets and mechanisms of neuroinflammation in neurodegeneration.

There are some limitations in the analysis. Through integrative and network analysis, we uncovered a set of core signaling pathways of neuroinflammation. However, first, the detailed signaling cascades among these neuroinflammation signaling genes in AD brain niche remain unclear. The general known signaling pathways in KEGG are not specifically designed for AD. For example, though the activation of T and NK cell signaling pathways can indicate the activation of some immune signaling–related proteins, the roles of these proteins in neuroinflammation remain unclear because the T and NK cells are not involved in the brain. In addition to the identified inflammation signaling pathways, it is interesting to further investigate the specific and detailed inflammation signaling cascades among the identified signaling targets that are potentially caused by the abnormal β-amyloid and tau protein aggregations. The other limitation is that the identified inflammation-related targets were not experimentally validated at the RNA or/and protein levels, such as qPCR or/and Western blotting/IHC. We will look for wet-lab collaborators for the potential experimental validations. Also, different brain regions might have different dysfunctional signaling pathways or different levels of neuroinflammation. Moreover, the current RNA-seq datasets are bulk tissue based, including multiple cell types, which confounds the gene expression change. Therefore, cell type composition correctness might affect the differential expression analysis. In addition, the neuroinflammation signaling pathways are reported to be affected by multiple cell types, such as microglia and astrocytes, in addition to neuron cells. Thus, it is important to analyze brain-region specific, single nuclear or cell RNA-seq data (Mathys et al., 2019) to uncover the finer-scaled neuroinflammation signaling pathways in different cell types and their signaling interactions in AD brain microenvironment and niche. To evaluate the relatively weak and strong inflammation levels, it is interesting to investigate the inflammation activation levels in control cases with different pathology levels measured by the clinical data. Some clinical information, such as beta-amyloid plaques, cerebral amyloid angiopathy, or/and tau pathologies in the brains of AD patients, of the two publicly available datasets were not available, thus some signaling targets and pathways, like HIF-1 signaling pathway, might be associated with subtypes of AD patients with vascular dementia. Therefore, it is interesting and critical to differentiate patients with vascular dementia from AD patients.

## Data availability statement

Publicly available datasets were analyzed in this study. This data can be found here: https://adknowledgeportal.synapse.org/Explore/Studies?Study=syn3219045, https://www.synapse.org/#!Synapse:syn23277389, and https://www.synapse.org/#!Synapse:syn3191087.

## Author contributions

Project was conceived by FL and CC. Transcriptomics datasets of AD samples were collected and pre-analyzed by AE, CS, OH, and CC. Methodology was developed and data were analyzed by FL. Results were discussed by FL, CC, AE, OH, EM, CK, and PP. The manuscript was prepared by FL and revised by CC, AE, WB, GW, CX, PP, EM, CK, and OH. All authors contributed to the article and approved the submitted version.

## Footnotes

1
https://www.synapse.org/#!Synapse:syn23277389

2
https://www.synapse.org/#!Synapse:syn3191087

3
https://www.synapse.org/#!Synapse:syn5550404

4
http://broadinstitute.github.io/picard/

5
https://www.gencodegenes.org/human/releases.html

6
https://www.niagads.org/igap-rv-summary-stats-kunkle-p-value-data
