# Supplementary material for: Weakly activated core neuroinflammation pathways were identified as a central signaling mechanism contributing to the chronic neurodegeneration in Alzheimer’s disease
Source: Front Aging Neurosci. 2022 Sep 27;14:935279. doi: 10.3389/fnagi.2022.935279 (PMC9551568; doi:10.3389/fnagi.2022.935279)
Supplement: Supplementary file 1 [file Data_Sheet_1.docx]

**Supplementary Tables**

**Table S1:** Differentially expressed genes (DEGs) between AD and control samples in Mayo and ROSMAP datasets.

| **Fold change** | **P-value** | **# of DEGs**  **in Mayo** | **# of DEGs**  **in ROSMAP** | **# of Common DEGs**  **in Mayo and ROSMAP** |
| --- | --- | --- | --- | --- |
| >=2.0 | <=0.05 | 22 (up), 5 (down) | 0 (up), 0 (down) | 0 (up), 0 (down) |
| >=2.0 | <=0.1 | 22 (up), 5 (down) | 0 (up), 0 (down) | 0 (up), 0 (down) |
| >=1.5 | <=0.05 | 210 (up), 84 (down) | 30 (up), 5 (down) | 15 (up), 4 (down) |
| >=1.5 | <=0.1 | 210 (up), 86 (down) | 30 (up), 5 (down) | 15 (up), 4 (down) |
| >=1.25 | <=0.05 | 958 (up), 873 (down) | 487 (up), 123 (down) | 227 (up), 56 (down) |
| >=1.25 | <=0.1 | 962 (up), 883 (down) | 488 (up), 126 (down) | 230 (up), 64 (down) |
| >=1.1 | <=0.05 | 2457 (up), 3687 (down) | 2610 (up), 1752 (down) | 1009 (up), 604 (down) |
| >=1.1 | <=0.1 | 2609 (up), 3952 (down) | 2700 (up), 1783 (down) | 1120 (up), 689 (down) |

**Table S2**: Enriched Kegg signaling pathways using up-regulated genes.

| **Name** | **Genes** | **pValue** |
| --- | --- | --- |
| Focal adhesion | EGFR, ERBB2, HGF, PDGFRB, BCL2, BIRC3, ELK1, PGF, RAP1A, VEGFC, FLT1, FLT4, VAV3, TLN1, VASP, PXN, FYN, CAPN2, COL1A2, COL6A1, COL6A2, FN1, TNC, ITGA5, ITGB1, ITGB5, ITGB8, LAMA4, SPP1, THBS2, THBS4, ITGA10, MYL9, MYL12A, DOCK1, PARVA, CAV1, ITGA8 | 1.39E-05 |
| Mineral absorption | HEPH, MT1A, MT1E, MT1F, MT1G, MT1H, MT1M, MT1X, MT2A | 2.56E-05 |
| ECM-receptor interaction | COL1A2, COL6A1, COL6A2, FN1, TNC, ITGA5, ITGB1, ITGB5, ITGB8, LAMA4, SPP1, THBS2, THBS4, ITGA10, CD44, NPNT, ITGA8, SDC4 | 0.00017828 |
| Proteoglycans in cancer | HPSE2, PLCE1, EGFR, ERBB2, HGF, CDKN1A, ELK1, MRAS, IGF2, RRAS, TGFB1, VAV3, ITPR3, PXN, STAT3, WNT6, FZD7, FZD8, FZD9, COL1A2, FN1, ITGA5, ITGB1, ITGB5, SMO, DCN, CAV1, CD44, IQGAP1, MSN, EZR, SDC2, SDC4, TIMP3 | 0.00035749 |
| Cytokine-cytokine receptor interaction | CSF1, IL1R1, TGFB1, TGFBR2, TNFRSF1A, NGFR, CXCR4, GDF11, CTF1, CXCL1, IL2RG, IL4R, CXCL8, INHBB, CXCL10, IL20RA, CCL2, BMP6, BMP7, GDF15, LTBR, TNFRSF10B, IL3RA, TNFRSF10A, TNFRSF11B, IL13RA1, IL15RA, OSMR, IL1R2, IL18R1 | 0.00053537 |
| Hippo signaling pathway - multiple species | YAP1, WTIP, WWC1, WWTR1, LATS2, TEAD1, TEAD4, TEAD3, TEAD2 | 0.00055489 |
| Pathways in cancer | PLCB3, EGFR, ERBB2, HGF, PDGFRB, BCL2, CDKN1A, DLL4, RELA, BIRC3, FADD, RXRA, HES1, GADD45G, ELK1, MECOM, FLT3LG, IGF2, PGF, TGFB1, TGFBR2, VEGFC, FLT4, LPAR4, CXCR4, GLI3, NFKBIA, IL2RG, IL4R, CXCL8, NFKB2, EGLN3, STAT3, CDK2, FOXO1, AGT, CDKN2B, SP1, NFE2L2, LRP5, WNT6, FZD7, FZD8, FZD9, FN1, IL3RA, ITGB1, LAMA4, FOS, CASP7, LEF1, TCF7L1, NOTCH2, NOTCH3, GLI1, SMO, IL13RA1, IL15RA, PIM1, EPAS1, KIF7, PML, HEYL | 0.0007582 |
| PI3K-Akt signaling pathway | PCK1, NOS3, EGFR, ERBB2, HGF, PDGFRB, BCL2, CDKN1A, RELA, RXRA, CSF1, FLT3LG, ANGPT2, IGF2, PGF, GNG12, VEGFC, EPHA2, FLT1, FLT4, GNG5, NGFR, LPAR4, CREB3L2, IL2RG, IL4R, CDK2, SGK1, DDIT4, COL1A2, COL6A1, COL6A2, PHLPP1, FN1, TNC, IL3RA, ITGA5, ITGB1, ITGB5, ITGB8, LAMA4, SPP1, THBS2, THBS4, ITGA10, OSMR, ITGA8 | 0.0010985 |
| Viral protein interaction with cytokine and cytokine receptor | CSF1, TNFRSF1A, CXCR4, CXCL1, IL2RG, CXCL8, CXCL10, IL20RA, CCL2, LTBR, TNFRSF10B, TNFRSF10A, IL18R1 | 0.00185617 |
| AGE-RAGE signaling pathway in diabetic complications | NOS3, PLCD3, PLCE1, PLCB3, PLCD1, BCL2, RELA, TGFB1, TGFBR2, VEGFC, CXCL8, CCL2, STAT3, FOXO1, AGT, COL1A2, ICAM1, PIM1 | 0.00213956 |
| Epstein-Barr virus infection | BCL2, CDKN1A, RELA, FADD, HES1, GADD45G, TAB2, IRAK1, MYD88, MAP2K3, NFKBIA, CXCL10, NFKB2, STAT3, CDK2, TNFAIP3, HLA-C, HLA-E, CD44, ICAM1, CD58, TAP1, B2M, SAP30 | 0.00560455 |
| MAPK signaling pathway | EGFR, ERBB2, HGF, PDGFRB, RELA, GADD45G, CSF1, DUSP1, ELK1, MECOM, MRAS, TAB2, FLT3LG, GNA12, ANGPT2, MKNK2, HSPA2, IGF2, IL1R1, IRAK1, MYD88, PGF, GNG12, MAP2K3, RAP1A, RRAS, TGFB1, TGFBR2, TNFRSF1A, VEGFC, MAPKAPK3, DUSP16, MAPKAPK2, EPHA2, FLT1, FLT4, NGFR, NFATC3, NFKB2, FOS, HSPB1 | 0.00589871 |
| Adipocytokine signaling pathway | PCK1, ACSBG1, ACACB, ACSL5, RELA, PPARA, RXRA, TNFRSF1A, NFKBIA, STAT3, ADIPOR2, SOCS3, SLC2A4 | 0.00604817 |
| Basal cell carcinoma | CDKN1A, GADD45G, GLI3, WNT6, FZD7, FZD8, FZD9, LEF1, TCF7L1, GLI1, SMO, KIF7 | 0.0076605 |
| NF-kappa B signaling pathway | BCL2, RELA, BIRC3, TAB2, IL1R1, IRAK1, MYD88, TNFRSF1A, NFKBIA, CXCL1, CXCL8, LTBR, NFKB2, TNFAIP3, ICAM1 | 0.00850971 |
| Regulation of actin cytoskeleton | EGFR, PDGFRB, MRAS, GNA12, GNG12, RRAS, VAV3, LPAR4, CXCR4, GNA13, PXN, FN1, ITGA5, ITGB1, ITGB5, ITGB8, ITGA10, MYL9, LIMK2, MYL12A, DOCK1, WASF2, IQGAP1, MSN, EZR, SPATA13, GIT1, DIAPH3, ITGA8, ARHGEF6 | 0.00867475 |
| IL-17 signaling pathway | RELA, TAB2, NFKBIA, FOS, FOSL1 | 0.01044254 |
| Hippo signaling pathway | TGFB1, TGFBR2, PARD3, BMP6, BMP7, WNT6, FZD7, FZD8, FZD9, LEF1, TCF7L1, YAP1, WTIP, AMOT, WWC1, WWTR1, LATS2, TEAD1, TEAD4, TEAD3, TP53BP2, TEAD2, SOX2 | 0.01316293 |
| TGF-beta signaling pathway | TGFB1, TGFBR2, INHBB, BMP6, BMP7, CDKN2B, SP1, BAMBI, DCN, LTBP1, SMAD6, NEO1, RGMA, TGIF2, TGIF1 | 0.01370407 |
| Human T-cell leukemia virus 1 infection | CDKN1A, CDKN2C, RELA, ELK1, IL1R1, TGFB1, TGFBR2, TNFRSF1A, NFATC2, NFATC3, NFKBIA, CREB3L2, IL2RG, LTBR, NFKB2, CDK2, CDKN2B, CHEK2, FOS, HLA-C, HLA-E, FOSL1, ICAM1, IL15RA, IL1R2, B2M | 0.01881732 |
| Complement and coagulation cascades | PLAT, SERPINA5, SERPINA1, CFB, TFPI, SERPING1, C1R, C4A, C4B | 0.02140549 |
| Staphylococcus aureus infection | ICAM1, CFB, C1R, C4A, C4B, CFH, CFI | 0.02493939 |
| Axon guidance | RRAS, EPHA2, PARD3, CXCR4, NFATC2, NFATC3, BMP7, FYN, ITGB1, MYL9, SMO, NEO1, RGMA, EFNB1, EPHB4, UNC5B, FES, RHOD, LIMK2, SRGAP1, RGS3, SEMA3F, BOC, NTN1, MYL12A | 0.02664014 |
| Insulin resistance | PCK1, ACACB, NOS3, PYGL, PYGM, RELA, PPARA, TNFRSF1A, NFKBIA, CREB3L2, STAT3, FOXO1, SOCS3, PPP1R3C, PPP1R3D | 0.02834387 |
| Human papillomavirus infection | ATP6V0E1, EGFR, PDGFRB, CDKN1A, RELA, FADD, HES1, TNFRSF1A, PARD3, CREB3L2, PXN, CDK2, FOXO1, WNT6, FZD7, FZD8, FZD9, COL1A2, COL6A1, COL6A2, FN1, TNC, ITGA5, ITGB1, ITGB5, ITGB8, LAMA4, SPP1, THBS2, THBS4, ITGA10, TCF7L1, NOTCH2, NOTCH3, MAML2, IRF1, ITGA8, HEYL | 0.02986106 |
| Breast cancer | EGFR, ERBB2, CDKN1A, DLL4, HES1, GADD45G, FLT4, NFKB2, SP1, LRP5, WNT6, FZD7, FZD8, FZD9, FOS, LEF1, TCF7L1, NOTCH2, NOTCH3, HEYL | 0.03748158 |
| Pertussis | RELA, IRAK1, MYD88, FOS, SERPING1, C1R, IRF1, C4A, C4B | 0.03748822 |
| Yersinia infection | RELA, TAB2, IRAK1, MYD88, MAP2K3, VAV3, NFATC2, NFATC3, NFKBIA, CXCL8, CCL2, PXN, FN1, ITGA5, ITGB1, FOS, DOCK1, WASF2 | 0.03974146 |
| NOD-like receptor signaling pathway | PLCB3, BCL2, RELA, BIRC3, FADD, TAB2, MYD88, ITPR3, P2RX7, NFKBIA, CXCL1, CXCL8, CCL2, TNFAIP3, IFI16, ERBIN, TRIP6, GSDMD, CASP4 | 0.04009447 |
| Maturity onset diabetes of the young | GCK, HES1, PAX6 | 0.0412998 |
| Pathogenic Escherichia coli infection | RELA, FADD, TAB2, GNA12, IL1R1, IRAK1, MYD88, TNFRSF1A, LPAR4, GNA13, NFKBIA, CXCL8, TNFRSF10B, TUBA1C, FOS, CASP7, TNFRSF10A, WASF2, EZR, CASP4, MYO1C, MYO10 | 0.04297897 |
| TNF signaling pathway | RELA, BIRC3, FADD, MAP2K3, TNFRSF1A, NFKBIA, CREB3L2, FOS, CASP7, MLKL, IRF1, CEBPB | 0.04341918 |
| Apoptosis - multiple species | BCL2, BIRC3, FADD, TNFRSF1A, NGFR, CASP7 | 0.05318839 |
| Rap1 signaling pathway | PLCE1, PLCB3, EGFR, HGF, PDGFRB, CSF1, MRAS, ANGPT2, PGF, MAP2K3, RAP1A, RRAS, VEGFC, EPHA2, FLT1, FLT4, NGFR, VAV3, ADORA2A, ADORA2B, PRKD3, LPAR4, PRKD1, PARD3, TLN1, VASP, ITGB1 | 0.0571398 |
| Human cytomegalovirus infection | PLCB3, EGFR, CDKN1A, RELA, FADD, ELK1, GNA12, IL1R1, GNG12, TNFRSF1A, GNG5, ITPR3, CXCR4, GNA13, NFATC2, NFATC3, NFKBIA, CREB3L2, CXCL8, CCL2, PXN, STAT3, SP1, HLA-C, HLA-E, TAP1, B2M | 0.06026243 |
| Apoptosis | BCL2, RELA, BIRC3, FADD, GADD45G, TNFRSF1A, NFKBIA, TNFRSF10B, CAPN2, TUBA1C, IL3RA, CTSH, FOS, CASP6, CASP7, TNFRSF10A, PARP4 | 0.06582258 |
| Adherens junction | EGFR, ERBB2, TGFBR2, PARD3, FYN, LEF1, TCF7L1, NECTIN2, WASF2, YES1, IQGAP1 | 0.06689148 |
| Cellular senescence | CDKN1A, RELA, GADD45G, MRAS, MAP2K3, RRAS, TGFB1, TGFBR2, MAPKAPK2, NFATC2, NFATC3, CDK2, FOXO1, CDKN2B, CHEK2, CAPN2, TRAF3IP2, HLA-C, HLA-E | 0.06916583 |
| Sulfur metabolism | PAPSS2, SQOR, SELENBP1 | 0.07578511 |
| Malaria | HGF, MYD88, ICAM1 | 0.07578511 |
| Galactose metabolism | GALM, GCK, PFKP, PGM1, B4GALT1 | 0.08119728 |
| Glycosaminoglycan degradation | HYAL1, NAGLU, HPSE2, HYAL2 | 0.09045551 |
| Legionellosis | RELA, HSPA2, MYD88, NFKBIA, CXCL1, CXCL8, CASP7 | 0.09062831 |
| Leukocyte transendothelial migration | RAP1A, VAV3, CXCR4, PXN, ITGB1, MYL9, MYL12A, ICAM1, MSN, EZR, CDH5, PECAM1 | 0.09482316 |
| Gastric cancer | EGFR, ERBB2, HGF, BCL2, CDKN1A, RXRA, GADD45G, TGFB1, TGFBR2, CDK2, CDKN2B, LRP5, WNT6, FZD7, FZD8, FZD9, LEF1, TCF7L1 | 0.09507402 |
| HIF-1 signaling pathway | PFKP, PFKFB3, NOS3, EGFR, ERBB2, BCL2, CDKN1A, RELA, ANGPT2, MKNK2, FLT1, LTBR, EGLN3, STAT3 | 0.10088945 |
| Wnt signaling pathway | PLCB3, NFATC2, NFATC3, LRP5, WNT6, FZD7, FZD8, FZD9, BAMBI, PRICKLE3, LEF1, SFRP1, SFRP2, SOX17, TBL1X, TCF7L1, ZNRF3, RSPO3, FOSL1 | 0.10427239 |
| Human immunodeficiency virus 1 infection | BCL2, RELA, FADD, TAB2, IRAK1, MYD88, GNG12, MAP2K3, TNFRSF1A, GNG5, ITPR3, CXCR4, NFATC2, NFATC3, PXN, WEE1, FOS, HLA-C, HLA-E, LIMK2, TAP1, B2M | 0.1074797 |
| Steroid hormone biosynthesis | HSD17B7, AKR1C2, HSD11B1, HSD11B2, AKR1C3 | 0.10838427 |
| Starch and sucrose metabolism | GCK, PGM1, PYGL, PYGM, GYG2 | 0.10838427 |
| Glucagon signaling pathway | PCK1, PFKP, ACACB, PYGL, PYGM, PLCB3, PPARA, ITPR3, PHKA1, CREB3L2, FOXO1, SIK1B | 0.11785197 |
| Rheumatoid arthritis | FLT1, FOS, ICAM1 | 0.11921547 |
| Fluid shear stress and atherosclerosis | NOS3, BCL2, RELA, DUSP1, IL1R1, TNFRSF1A, CCL2, NFE2L2, FOS, PLAT, CAV1, ICAM1, CDH5, PECAM1, IL1R2, SDC2, SDC4 | 0.12180353 |
| Th17 cell differentiation | RXRA, IL1R1, TGFB1, TGFBR2, NFATC2, NFATC3, NFKBIA, IL2RG, IL4R, STAT3, FOS | 0.12754476 |
| Inflammatory bowel disease (IBD) | RELA, TGFB1, IL2RG, IL4R, STAT3, IL18R1 | 0.13209705 |
| Prostate cancer | EGFR, ERBB2, PDGFRB, BCL2, CDKN1A, RELA, NFKBIA, CREB3L2, CDK2, FOXO1, LEF1, TCF7L1 | 0.13483793 |
| Cushing syndrome | PLCB3, EGFR, CDKN1A, CDKN2C, RAP1A, ITPR3, CREB3L2, CDK2, AGT, CDKN2B, SP1, WNT6, FZD7, FZD8, FZD9, LEF1, TCF7L1 | 0.13563822 |
| Th1 and Th2 cell differentiation | DLL4, RELA, NFATC2, NFATC3, NFKBIA, IL2RG, IL4R, FOS, NOTCH2, NOTCH3, MAML2 | 0.1367585 |
| Natural killer cell mediated cytotoxicity | VAV3, NFATC2, FYN, TNFRSF10B, TNFRSF10A, HLA-C, HLA-E, ICAM1, ICAM2, MICA, KLRC3, SH3BP2, CD48 | 0.14101973 |
| Primary bile acid biosynthesis | CYP39A1, HSD3B7, ACOX2 | 0.14366887 |
| Small cell lung cancer | BCL2, CDKN1A, RELA, BIRC3, RXRA, GADD45G, NFKBIA, CDK2, CDKN2B, FN1, ITGB1, LAMA4 | 0.14380894 |
| Parathyroid hormone synthesis, secretion and action | PLCB3, EGFR, BCL2, CDKN1A, GNA12, ITPR3, GNA13, CREB3L2, SP1, LRP5, FOS, MMP14 | 0.15309099 |
| Ether lipid metabolism | CHPT1, PLPP1, PLPP3, PAFAH1B3, ENPP2, UGT8 | 0.16139156 |
| Arginine biosynthesis | NAGS, GLUL, NOS3, GPT2 | 0.16498016 |
| Notch signaling pathway | DLL4, HES1, NOTCH2, NOTCH3, MAML2, KAT2B, HEYL | 0.17719556 |
| MicroRNAs in cancer | EGFR, ERBB2, PDGFRB, BCL2, CDKN1A, STAT3, DDIT4, TNC, ITGA5, NOTCH2, NOTCH3, CD44, EZR, PIM1, TIMP3 | 0.18532428 |
| Fat digestion and absorption | PLPP1, PLPP3 | 0.19672574 |
| Systemic lupus erythematosus | C1R, C4A, C4B | 0.19672735 |
| Toxoplasmosis | BCL2, RELA, BIRC3, TAB2, HSPA2, IRAK1, MYD88, MAP2K3, TNFRSF1A, NFKBIA, STAT3, ITGB1, LAMA4 | 0.1974987 |
| Measles | BCL2, RELA, FADD, TAB2, IRAK1, MYD88, NFKBIA, IL2RG, STAT3, CDK2, FOS, TNFAIP3, MSN | 0.1974987 |
